# Curable Sexually Transmitted Infections Among a Population of South African Nonhealth Seeking Men Who Have Sex With Men Who Were Invited for Screening—A Brief Report

**DOI:** 10.1177/23259582241299468

**Published:** 2024-12-05

**Authors:** Matshidiso Adelaide Malefo, Olalekan Abdulwahab Ayo-Yusuf, Mathildah Mpata Mokgatle

**Affiliations:** 1 School of Public Health, 37715 Sefako Makgatho Health Sciences University, Pretoria, Gauteng, South Africa; 2 School of Health Systems and Public Health, 56410 University of Pretoria, Pretoria, Gauteng, South Africa

**Keywords:** men who have sex with men, sexually transmitted infections, brief report, South Africa

## Abstract

**Background:** This study assessed sexually transmitted infection (STI) results among nonhealth-seeking men who have sex with men (MSM) who had not previously screened for curable STIs. **Methods:** Secondary data analysis of a sample of 164 MSM who were STI infection naïve was performed. Data were collected in the Clinical Research Unit (MeCRU) among the MSM in the Tshwane North area, South Africa. **Results:** Over half of the sample (58.5%; *n* = 96) were in casual relationships, 81.7% (*n* = 134) had reported testing for HIV in the previous 3 months, and 68.9% tested positive for STIs. Logistic regression revealed that the odds of a positive STI test result were significantly higher among those who had reportedly tested for HIV in the previous 3 months compared to those who had not (OR = 2.43; 95% CI = 1.06-5.69). **Conclusion:** The study revealed a high prevalence of STI among nonhealth seeking MSM and STI diagnosis was associated with having tested for HIV in the previous 3 months. It is, therefore, important to offer regular STIs screening services to the MSM community.

## Background

Overall, the prevalence of curable bacterial sexually transmitted infections (STIs) among men who have sex with other men (MSM) in South Africa is a significant public health concern. The World Health Organization (WHO) has set a goal to eradicate STI epidemics by 2030 with targets including a 90% decrease in the incidence of *Treponema pallidum* (*T pallidum*) and *Neisseria gonorrhoeae* globally.^1^ However, in South Africa, MSM are disproportionately affected by STIs, including human immunodeficiency virus (HIV). The presence of STIs increases the risk of contracting and/or transmitting HIV infection.^2^ A metaanalysis has shown that MSM in low- and middle-income countries is 19.3 times more likely to be HIV-positive than the overall population. Risk factors for STI acquisition among MSM in South Africa include unprotected anal intercourse, having multiple sexual partners, and a lack of knowledge about safer sex practices.^3^ The findings of the retrospective data analysis collected from October 2016 to July 2021 by HIV Vaccine Trial 702 studies and Sibanye MSM HIV intervention prevention pilot study from 2015 to 2016, which also showed that bacterial STI burden was higher in MSM compared to non-MSM males (26.0% vs 14.3%, *P* = .001).^4,5^

The syndromic management approach is currently adopted in South African STI treatment guidelines. The syndromic management approach is when individuals recognize STI signs and symptoms, seek medical attention, and report the symptoms to healthcare professionals for effective management.^6^ The current guidelines^7,8^ recommends that sexually active MSM be tested for bacterial STIs at locations of sexual contact (the penis, anus, and oral) at least once a year, regardless of their condom use. However, there are several barriers to accessing STI testing and treatment of MSM in South Africa, including stigma and discrimination, a lack of targeted health education and outreach programs, and structural barriers such as the limited availability of services, long waiting times, a lack of confidentiality and privacy, and high costs.^3,9–12^

Lack of access to testing and treatment services could lead to the spread of STIs, including anti-microbial resistance, and could also increase the risk of HIV transmission. It is important to note that even though chlamydia, gonorrhea, and syphilis, which are bacterial STIs, and trichomoniasis, which is a parasitic STI, can be cured with existing medication regimens, drug resistance may jeopardize their susceptibility to treatment.^13^

One of the objectives of the study was to assess the prevalence of curable STIs among the MSM in a province of South Africa. This article further explored the data of a subsample of STI naïve MSM, that had reported to not have had STI testing or treatment in a period of three months prior to enrolling to the study.

## Method

### Study Design and Study Sample

The primary baseline data collection which has been previously described^14^ involved a cross-sectional survey, where researchers examined asymptomatic MSM living in and around Tshwane North District, South Africa. Data were collected from December 2021 to May 2022. However, for the purpose of the current study, samples were restricted to participants who had reported not to have ever had STI treatment nor tested for STI within the three months prior to the survey (*n* = 164).

### Recruitment

Participants were recruited using numerous strategies including outreach in the streets, dance clubs, bars, and health clubs as well as through community forums and snowballing. Only participants who identified themselves as MSM were recruited and enrolled. The details of the recruitment strategy have been previously published.^14^

### Data Collection and Measures

Baseline data was collected through a self-administered questionnaire that included information on the participants’ demographics, sexual behavior, and history of recent STI treatment in the 3 months before joining the study. All study participants were then offered STI screening. A trained clinician used direct swabbing to obtain samples for testing from rectal, oral, genital swabs, and blood samples were also collected to test syphilis. The samples were sent to the laboratory. The participants were released to allow for the laboratory turnaround time of one week, and then the results were sent to the participants together with the referral via home visits, while some returned to the facility to collect the results personally. Participants were encouraged to go to their nearest local clinics with their laboratory results for further care, as the case may be. Participants were re-imbursed R150 for transportation costs, their time, and efforts. *Neisseria gonorrhoeae* (NG), *Chlamydia trachomatis* (CT), *Mycoplasma genitalium and homonis* (MG & MH), *Trichomonas vaginalis* (TV), *Ureaplasma urelyticum and parvum* (UU & UP), *Treponema pallidum* (TPHA), and *Syphilis* were all tested for in each of the participants. In the current study, a positive curable STI result was recorded for anyone testing positive for anyone of these bacterial infections. The data were kept in a secure place and follow-up visits were scheduled according to the participants first date of enrollment.

### Data Analysis

The data were analyzed using Stata 17.0 software. Descriptive statistics were used to summarize the data and, data were categorized and presented as frequencies. Chi-square statistics or the nonparametric equivalent—Fischer's exact test, was used to determine if there were statistically significant differences between 2 or more groups of independent variables (known partner HIV status, sexual practice, etc) and binary dependent variable (tested positive for STI or not). Adjusted odd ratios were used to determine the independent relationship between these potential independent variables and the main outcome/dependent variable—having tested positive for STI.

## Results

The results of this study focused on the subsample of 164 MSM who reported not having had any STI treatment or testing in the previous 3 months prior to enrolling in the study. The article is reporting on the prevalence and factors associated with subsequently testing positive for STI.

About 58.5% (*n* = 96) of the MSM reported that they had completed high school or a higher level of education, while 51.2% did not have a source of income. The majority (58.5%) of the MSM had being in a casual relationship and 42.2% did not know their last sex partner’s HIV status. According to their sexual practices, 70% practice a combination of anal sex, oral sex, and other methods whereas 20.2% practice anal insertive sex. Other sexual behaviors and practices are depicted in Table 1. As compared to those who had not, the odds of a positive STI test result were significantly higher among those who had tested for HIV in the previous 3 months (OR = 2.43; 95% CI = 1.06-5.69) (Table 2).

**Table 1.** Socio-Behavioral Characteristics Sample of MSM (*n* = 164).

Variables	Frequencies % (*n*)
Education level	
> High school	41.5% (*n* = 68)
High school	54.4% (*n* = 86)
< High school	6.1% (*n* = 10)
Employment status	
Unemployed/no income	51.2% (*n* = 84)
Student	10.4% (*n* = 17)
Employed/income	38.4% (*n* = 63)
Type of relationship	
Casual	58.5% (*n* = 96)
Stable partner	41.5% (*n* = 68)
Sexual orientation	
Gay	64% (*n* = 105)
Bisexual	25% (*n* = 41)
Other (eg, heterosexuals)	11% (*n* = 18)
Number of sex partners	
One	45.1% (*n* = 74)
Two	26.8% (*n* = 44)
Three or more	28.0% (*n* = 46)
Sexual practice	
Insertive anal sex	20.2% (*n* = 33)
Receptive anal sex	9.2% (*n* = 15)
Combination, oral, other	70.6% (*n* = 115)
Prevention method during sex	
None/withdrawal	9.8% (*n* = 16)
Only used lubricant	19.5% (*n* = 32)
Use condoms with or without lubricant	70.7% (*n* = 116)
Last partner's HIV status	
No response	4.9% (*n* = 8)
Positive	40.2% (*n* = 66)
Negative	12.8% (*n* = 21)
Do not know	42.1% (*n* = 69)
Had sex under the influence of alcohol	
Yes	36% (*n* = 59)
No	64% (*n* = 105)

Abbreviations: STI, sexually transmitted infection; MSM, men who have sex with men; HIV, human immunodeficiency virus.

**Table 2.** Characteristics of MSM Diagnosed With Curable Bacterial STIs Out of All Those Who Reported No Testing or Treatment Visit in Previous 3 Months (*n* = 164).

Variables	STI diagnosis (*n* = 113) % (*n*)	*P*-value		^a^OR (95% CI)
Education level					
> High school	63.2 (43)	.271		
High school	74.4 (64)			
< High school	6.0 (6)			
Source of income					
No income	63.1 (53)	.196		
Student	82.4 (14)			
Income/salary/partner	73.0 (46)			
Relationship status					
Casual	66.7 (64)	.462		
Stable	72.1 (49)			
Last sex partner					
Both male and female	65.5 (38)	.488		
Male	70.8 (75)			
Sex orientation					
Heterosexual	61.1(11)	.432		
Bisexual	63.4 (26)			
Gay	72.4 (76)			
Number of partners					
One	63.5 (47)	.379		
Two	75.0 (33)			
Three or more	71.7 (33)			
Sex practice					
Insertive anal sex	60.6 (20)	.054		1
Receptive anal sex	46.7 (7)			0.68 (0.19-2.41)
Combination, oral, other (eg, rimming bottom or rimming top)	73.9 (85)			1.83 (0.80-4.17)
Condom use					
None/withdrawal	62.5 (10)	.639		
Lubricant only	75.0 (24)			
Condomize (male/female)	68.1 (79)			
Tested for HIV					
No	50.0 (15)	.013		1
Yes	73.1 (98)			2.43 (1.06-5.60)
Last partner's HIV status					
No response	62.5 (5)	.968		
Negative	69.7 (46)			
Positive	71.4 (15)			
Do not know	68.1 (67)			
Had sex under alcohol influence					
Yes	67.8 (40)	.819		
No	69.0 (73)			

Abbreviations: STI, sexually transmitted infection; MSM, men who have sex with men; HIV, human immunodeficiency virus.

^a^ OR (95% CI) = adjusted odds ratio (95% confidence interval) from a multivariable-adjusted binary regression model.

## Discussion

This study reports on the prevalence of curable STIs among MSM who reported not to have had any STI tested or treated in the past 3 months, and we found as high as 68.9% tested positive for STI after screening. Hence the a need to predict or identify risk for STI among these asymptomatic MSM. Similar to other studies, it would appear that in the absence of symptoms, the infected participants in this study were not persuaded to seek health services or change their risk practices, and therefore increasing their risk of HIV transmission and acquisition.^15–17^

Our study reiterates that the high burden of STIs among sexually active asymptomatic MSM is of significant public health importance. Furthermore, the high prevalence of STI in this study demonstrates the value of testing multiple anatomic sites to avoid infections that would have remained undetected among asymptomatic MSM

This study showed that in a controlled regression model, the only significant predictor for testing positive for STI was having previously tested for HIV also in the previous 3 months. The sex practice was marginally significant in the bivariate analysis with those engaging in a combination of various other sexual practices tending to be most at risk and those practicing only receptive anal sex being least at risk. However, after controlling for testing for HIV, the sexual practice was no longer significant. This suggests the association between sexual practices and testing positive for STI was possibly mediated by a general feeling of engaging in risky sexual behaviors and hence having to go test for HIV. The implication is that all MSM that come for HIV testing should be always screened for STIs as there are over 2-fold higher odds they may test positive for other STIs irrespective of their result. This finding suggests that there is a need for public health efforts directed towards MSM to emphasize screening for curable STIs other than just HIV. Other effective interventions to increase testing and treatment uptake among MSM include offering free, low-cost testing, increasing the availability of testing sites, and outreach to inform MSM of the importance of regular testing for STIs.

## Limitation

Our study has limitations. We enrolled a convenience sample at a single site, and the data may not be generalizable to MSM in South Africa as a whole. Another limitation of our study was the relatively small sample size, which might have limited the statistical power to detect statistical associations during the analysis of the data (ie, possible type II error). Despite the limitations of this study, it represents one of the few such published studies from resource-poor settings in a population of MSM and provides important practice and policy implications.

## Conclusion

The MSM that reported no prior STI treatment or screening in this study had a high prevalence of STI, which is associated with having recently tested for HIV. It is important to increase STI screening and/or testing, particularly at the same time MSM report for HIV testing. This opportunistic screening for STIs may require increasing access to sexual health clinics and should be combined with providing education and resources on safe sexual practices and addressing the social determinants, such as stigma and discrimination, that contribute to the vulnerability of MSM to STIs.
